# The Royal Infirmary, Sunderland

**Published:** 1912-04-06

**Authors:** Frank W. Melvin

**Affiliations:** Resident Medical Officer.


### THE EDITOR'S LETTER-BOX.

THE ROYAL INFIRMARY, SUNDERLAND.

Sir,—In The Hospital of March 23 Sir Henry Burdett publishes his report on the Royal Infirmary, Sunderland. I was sorry to read that his opinion of the institution was so unfavourable, but was pleased to see the praise he gave to the pharmacist in his last sentence; it relieved one to think that there was one small oasis left in the desert of chastisement meted out to the Sunderland Infirmary. His statement relating to the number of deaconesses working at the Infirmary is erroneous, for in 1873, when they were first appointed, seven were employed, and in 1889, when the nursing school was founded, there were twenty, two of whom still remain with us.

No one is more alive to the fact that the central administration block needs entire reconstruction than myself, but until that is done the work must be carried on under the existing arrangements, and I think for the Governors to expend the available capital of £30,000 to attain that object would be an act of folly from which the infirmary would never recover, when one considers that for a number of years past the balance sheet has shown a deficit.

I am sorry that Sir Henry Burdett made no mention in his report of the condition of the patients and the cleanliness of the wards, beds, corridors, etc., and I fail to agree with him that there is "an air of considerable slackness in the general control." No one who is acquainted with this Infirmary and the managing sister can for one moment accuse her of being slack; if anything, she reaches the other extreme in her enthusiasm, as many of the past and present staff can testify. A hospital gate-porter of real ability is so seldom met with that the appointment of a liveried person with the average ability of his class would not increase the facility of working the entrance, but rather the expense.

The Royal Infirmary, Sunderland, will compare extremely favourably with any provincial hospital having over 100 beds. To the staff residing and working in the infirmary under the present conditions is due no small amount of credit, instead of the undeserved censure, when one considers the number of occupied beds to each nurse and the average number of in-patients per bed occupied; also to the management, when one considers the average cost per bed occupied on the total ordinary expenditure.

With regard to the Children's Hospital, it is very evident that only a certain proportion of the large number of children residing within the area of the Sunderland Infirmary will partake of the immense good to be derived there, unless the age-limit is reduced, seeing that there is accommodation for only fifty-five patients, whereas the Royal Infirmary could accommodate over seventy.

Yours, etc.,

Frank W. Melvin,

Resident Medical Officer.

[We have great pleasure in publishing Dr. Melvin's letter, but a careful perusal of it and of our report will show that he has not dealt with most of the points raised, except that relating to the reconstruction of the central block, which he admits is necessary.—Ed. The Hospital.]

